# Prevalence of Coronary Endothelial and Microvascular Dysfunction in Women with Symptoms of Ischemia and No Obstructive Coronary Artery Disease Is Confirmed by a New Cohort: The NHLBI-Sponsored Women's Ischemia Syndrome Evaluation–Coronary Vascular Dysfunction (WISE-CVD)

**DOI:** 10.1155/2019/7169275

**Published:** 2019-03-11

**Authors:** R. David Anderson, John W. Petersen, Puja K. Mehta, Janet Wei, B. Delia Johnson, Eileen M. Handberg, Saibal Kar, Bruce Samuels, Babak Azarbal, Kamlesh Kothawade, Sheryl F. Kelsey, Barry Sharaf, Leslee J. Shaw, George Sopko, C. Noel Bairey Merz, Carl J. Pepine

**Affiliations:** ^1^University of Florida, Gainesville, Florida, USA; ^2^Emory University School of Medicine, Atlanta, Georgia, USA; ^3^Smidt Heart Institute, Cedars-Sinai Medical Center, Los Angeles, California, USA; ^4^University of Pittsburgh, Pittsburgh, Pennsylvania, USA; ^5^Rhode Island Hospital, Providence, Rhode Island, USA; ^6^Cardiovascular Outcomes Research and Epidemiology, Emory University, Atlanta, Georgia, USA; ^7^National Institutes of Health/National Heart, Lung, and Blood Institute, Bethesda, Maryland, USA

## Abstract

**Objective:**

In a separate, contemporary cohort, we sought to confirm findings of the original Women's Ischemia Syndrome Evaluation (WISE).

**Background:**

The original WISE observed a high prevalence of both invasively determined coronary endothelial and coronary microvascular dysfunction (CMD) that predicted adverse events in follow-up.

**Methods:**

We comparatively studied the WISE-Coronary Vascular Dysfunction (CVD) cohort (2009-2011), with signs and symptoms of ischemia but without significant CAD, to the original WISE (1997-2001) cohort. CMD was defined as coronary flow reserve (CFR) ≤2.5, or endothelial dysfunction as epicardial coronary artery constriction to acetylcholine (ACH), or <20% epicardial coronary dilation to nitroglycerin (NTG).

**Results:**

In WISE (n=181) and WISE-CVD (n=235) women, mean age in both was 54 years, and 83% were white (WISE) vs 74% (WISE-CVD, p=0.04). Use of hormone replacement therapy was less frequent in WISE-CVD vs WISE (46% vs 57%, p=0.026) as was presence of hypertension (40% vs 52%, p=0.013), hyperlipidemia (20% vs 46%, p<0.0001), and smoking (46% vs 56%, p=0.036). Similar rates were observed in WISE-CVD and WISE cohorts for CMD (mean CFR 2.7±0.6 vs 2.6±0.8, p=0.35), mean change in diameter with intracoronary ACH (0.2±10.0 vs 1.6±12.8 mm, p=0.34), and mean change in diameter with intracoronary NTG (9.7±13.0 vs 9.8±13.5 mm, p=0.94), respectively.

**Conclusions:**

This study confirms prevalence of CMD in the contemporary WISE-CVD cohort similar to that of the original WISE cohort, despite a lower risk factor burden in WISE-CVD. Because these coronary functional abnormalities predict major adverse cardiac events, clinical trials of therapies targeting these abnormalities are indicated.

## 1. Introduction

The presence of coronary microvascular abnormalities in patients presenting with symptoms and/or signs of ischemia but without obstructive coronary macrovascular disease is increasingly recognized. This finding appears to be more frequent in women than in men [1]. As many as 30-65% of women undergoing angiography to evaluate angina are found to have no obstructive coronary artery disease (CAD) [2–4]. Even among patients with acute coronary syndromes, the absence of obstructive CAD (< 50% stenosis) occurs in approximately 7-32% of women compared with only 6-12% of men [5–7]. We and others have demonstrated that many of these women have coronary microvascular dysfunction (CMD) [8]. CMD appears to be an early manifestation of atherosclerosis and is a predictor of future cardiovascular events including death, myocardial infarction (MI), hospitalization for unstable angina or heart failure, and stroke [9–16].

The National Heart, Lung, and Blood Institute (NHLBI)-sponsored Women's Ischemia Syndrome Evaluation (WISE) was designed to improve diagnostic evaluation and understanding of mechanisms underlying ischemic heart disease (IHD) in women [11]. The original cohort enrolled from 1997 to 2001 and was characterized by a high frequency (~65%) of no or nonobstructive CAD in women presenting with symptoms and signs of chronic IHD. An intravascular ultrasound (IVUS) substudy of WISE found that evidence for atherosclerotic plaque was present in >80% of these women with so-called “normal” angiograms [10]. Subclinical CAD has also been found in 85% of patients over 50 years of age by IVUS in another study [17]. Additional studies also led to the recognition that many of these patients had abnormal coronary vascular reactivity. There was a high prevalence of abnormal endothelial-dependent response to acetylcholine (ACH), as well as an abnormal coronary flow reserve in response to adenosine. Both of these vascular function abnormalities were associated with increased risk for adverse events in follow-up [13, 16]. Those patients with the most abnormal coronary flow response had the highest adverse event rate during follow-up [18].

The contemporary NHLBI-sponsored WISE-Coronary Vascular Dysfunction (WISE-CVD) cohort enrolled subjects from 2009-12 to further evaluate women with symptoms and signs of IHD but exclusively without obstructive CAD (<50% stenosed). This subsequent cohort of patients, with similar inclusion criteria as in the original group, except limited to only those without obstructive CAD, allows comparison of the prevalence of abnormal coronary vasomotion in women with symptoms and signs of ischemia but without obstructive CAD.

## 2. Methods

The original WISE was an NHLBI-sponsored four-center study assessing cardiovascular function using state-of-the-art techniques in women who were referred for coronary angiography to evaluate symptoms and signs of IHD. The protocol and results have been described in detail elsewhere [11]. Coronary reactivity testing (CRT) was performed at the University of Florida (Gainesville, FL), University of Pittsburgh (Pittsburgh, PA) and Allegheny General Hospital (Pittsburgh, PA) centers. In the contemporary WISE-CVD cohort, CRT assessment was done at Cedars-Sinai Medical Center (Los Angeles, CA) and the University of Florida. Ethics committees at participating institutions approved all WISE protocols and all study participants provided written informed consent. Demographic data were recorded using standardized WISE questionnaires. For both cohorts, the coronary angiograms and coronary reactivity data were assessed using similar criteria at independent core labs (Brown University, Providence, RI, and the University of Florida, Gainesville, FL, respectively) masked to all other data.

### 2.1. Assessment of Coronary Vascular Function

All vasoactive medications were withheld prior to CRT as follows: calcium antagonists for ≥48 hrs; nitrates, beta blockers, angiotensin converting enzyme inhibitors or angiotensin receptor blockers, and caffeine ≥24 hours; short-acting nitrates and/or nicotine ≥4 hrs. Diagnostic coronary angiography was performed via the femoral approach with standard diagnostic catheters. CRT was performed in the left anterior descending (LAD) as the preferred artery, the left circumflex was used if the LAD could not be safely accessed. A Doppler-tipped guidewire (FloWire, JOMED/Cardiometrics, Mountain View, CA, and subsequently ComboMap in some WISE and all WISE-CVD patients, with the Volcano®FloWire 300 cm, San Diego, CA) was used to measure coronary flow velocity. Coronary flow reserve (CFR), as an index of endothelial-independent microvascular function, was measured with adenosine (Adenocard, Fujisawa USA) [13]. Intracoronary boluses of 18 mcg only (WISE cohort) or 18 and 36 mcg (WISE-CVD cohort) were administered. A saline flush followed each dose of adenosine. CFR was calculated as the ratio of average peak velocity (APV) during hyperemia to APV at baseline. Subsequent CFR measurements were made after the APV returned to baseline. A CFR of ≤2.5 was considered abnormal in both cohorts.

Assessment of endothelial function was performed following the protocol of intracoronary infusion of acetylcholine (ACH). ACH (Miochol-E, CibaVision) was administered IC over three minutes. Doses of 0.182 mcg/ml (10^−6^ mol/L) = 0.364 mcg, 1.82 mcg/ml (10^−5^ mol/L) = 3.64 mcg, and 18.2 mcg/ml (10^−4^ mol/L) = 36.4 mcg were used in the WISE cohort. The 0.182 mcg/ml (0.364 mcg) and 18.2 mcg/ml (3.64 mcg) doses were used in the WISE-CVD cohort. A Doppler measurement of APV was obtained with each ACH infusion. Subsequent infusions were performed after the APV had returned to baseline. Following each infusion cine-images were obtained for quantitative coronary angiography (QCA). Measurement of vessel diameter was taken 5 mm distal to the tip of the Doppler wire. A normal endothelial response to ACH was defined as any vasodilatation. An abnormal response was vasoconstriction or a lack of vasodilatation. Lastly, we performed angiography after IC nitroglycerin and used QCA in both groups to assess for coronary vasodilatation.

Serial angiography was performed in the same views. All vessel diameter measurements were made at 5 mm distal to the tip of the Doppler wire. For successive diameter measurements, the same vessel segment was used. All recordings were analyzed at a core laboratory masked to all other patient data. Hemodynamic parameters were recorded before, during, and after each of the adenosine, ACH, and NTG infusions.

### 2.2. Statistics

The baseline characteristics of the study participants are summarized using means and standard deviations for continuous variables. Percentages were used for categorical variables. The data were checked for distribution normality, and differences between CFR, changes in vessel diameter following ACH, and changes in diameter after NTG were assessed. A t-test was used to assess any differences between these variables in the old versus the new cohort. All tests were two-sided, and a p<0.05 was considered statistically significant. Univariate predictors of diameter change with CFR, ACH, and NTG were assessed. Multivariate modeling was then performed with univariate predictors that were p < 0.20. All analyses were performed with SAS software version 9.3 (SAS Institute).

## 3. Results

There were 181 women who underwent CRT in the original WISE cohort and 235 in the subsequent WISE-CVD cohort. The mean age was 54 years in both groups (Table 1). There were fewer Caucasian women in the latter group (74 vs. 83%, p =0.04). Levels of education were higher in the later WISE-CVD group, while cardiac risk factors were less prevalent in the later cohort, and hormone replacement therapy use was less frequent in the contemporary group.

**Figure 1:**
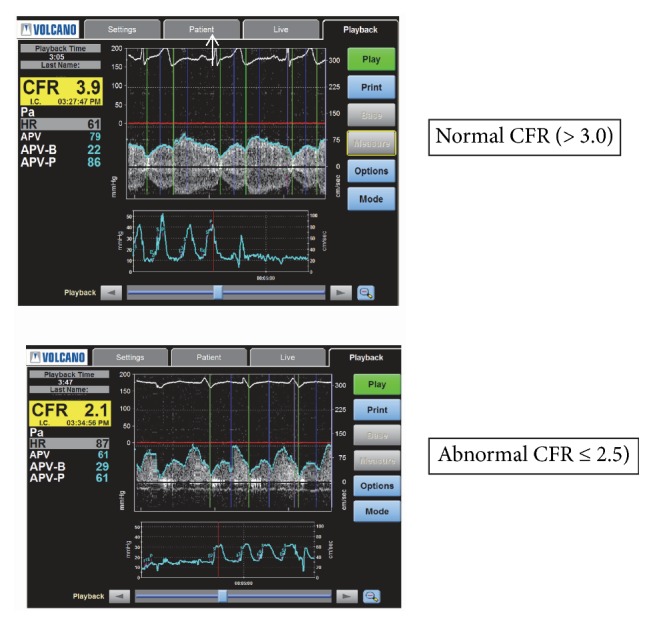
Example of a normal and abnormal coronary flow reserve (CFR) measurement.

**Figure 2:**
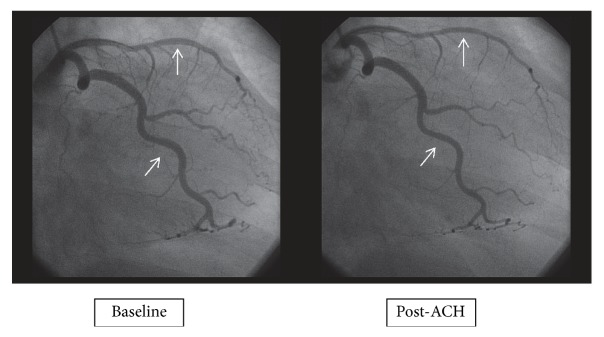
Angiography before and after the administration of intracoronary acetylcholine (ACH).

Mean CFR measured in WISE-CVD was not different than that in the WISE cohort (2.7 ± 0.6 versus 2.6 ± 0.8, p=0.35, Table 2). The percentage of patients with a CFR ≤2.32, a cut point previously identified as predicting adverse events [18], was also not significantly different between the two groups (31 vs 36%, p= 0.27).  Figure 1 shows an example of a woman with normal and another with abnormal CFR in response to IC adenosine. In the WISE-CVD cohort, the percentage of women who had an abnormal response to IC ACH was not different compared to the early cohort (54 vs 44, p = 0.10). The mean change in vessel diameter following IC ACH was also not different, nor was the endothelial-independent mean change in vessel diameter in response to IC NTG (Table 2).  Figure 2 provides an example of an abnormal endothelial-dependent response to IC ACH. Finally, the endothelial-independent mean change in vessel diameter in response to IC NTG was not different between the original and the contemporary cohorts (9.7 ± 13.0 vs 9.8 ± 13.5, p = 0.94, Table 2).

In a multivariate model, a history of hypertension (-0.18, p = 0.016) and beta blocker use (-0.21, p = 0.016) predicted a lower CFR, while statin use (0.19, p = 0.028) was associated with a higher CFR. There were no multivariate predictors of the diameter response to ACH or NTG. For both of these variables, older patients in the later WISE-CVD cohort tended to have less vasoconstriction to ACH (WISE 0.15, p=0.24, WISE-CVD -0.16, p=0.022), and less vasodilatation to NTG (WISE 0.13, p=0.31, WISE-CVD -0.17, p=0.048).

**Figure 3:**
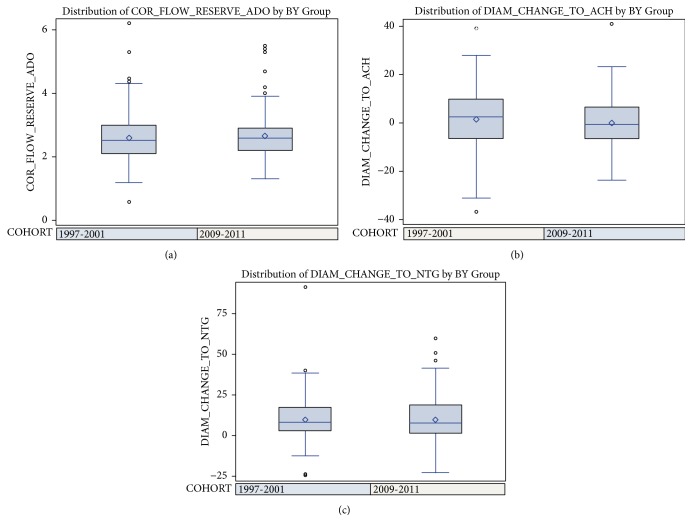
Comparison of (a) coronary flow reserve (CFR), (b) diameter change to acetylcholine (ACH), and (c) diameter change to nitroglycerin (NTG) according to the original WISE and later WISE-CVD cohorts.

Figure 3 provides a comparison of the three coronary reactivity variables between the early WISE population from 1997-2001 (n=181) and the later WISE-CVD group of women from 2009-2011 (n=235).

## 4. Discussion

In the original WISE cohort, we observed a high prevalence of coronary vascular function abnormalities in women presenting with signs and symptoms of ischemia but without significant CAD. Nearly half of these women had an abnormal CFR, just less than half had an abnormal response to ACH, and over three quarters had an abnormal response to NTG. In the current WISE-CVD cohort, we observed a very similar abnormal CFR prevalence (40%). Furthermore, the degree of both endothelial-dependent and independent coronary vascular dysfunction was very similar. Our report is the first one showing consistent abnormalities of coronary reactivity between two separate time periods and populations of women characterized by an ischemic presentation but without significant epicardial coronary artery disease. This similar prevalence occurred despite enrolling women with fewer traditional risk factors in the WISE-CVD cohort. This finding suggests that the functional abnormalities identified by coronary reactivity study are not necessarily explained by traditional risk factors, as we have previously described [19]. The presence of a similar prevalence of coronary vascular dysfunction in a contemporary cohort of women suggests that, in fact, this disorder remains prevalent among this population of women. This similar prevalence and severity is noteworthy, as patients from the original WISE cohort have now been followed now for over 10 years. Patients with no or nonobstructive CAD have been shown to experience a higher event rate than originally anticipated [18]. Compared to age-matched reference women without manifest disease or ischemic symptoms, WISE women were found to have an increased cardiovascular event rate [20].

There are currently no evidence-based guidelines for treatment of patients with CMD in the absence of obstructive CAD. In a meta-analysis of randomized controlled trials, patients with no obstructive CAD treated with angiotensin converting enzyme inhibitors (ACE-Is) had improvement in endothelial function measured by brachial artery flow-mediated dilation [21]. In a randomized, placebo controlled trial of women without obstructive CAD from the initial WISE cohort, those receiving ACE-I (quinapril 80 mg/d) had greater improvement in CFR over 16 weeks compared with placebo [22]. Additionally, ACE-I therapy reduced angina and this response was linked with greater improvement in CFR. This effect appeared most prominent in those women with the lowest baseline CFR [22]. Several additional shorter-term trials of ACE-I have found similar benefits relative to angina reduction and improvement in other markers of vascular function.

Use of traditional antianginal and antiatherosclerosis medications and some novel agents may be beneficial; however clinical trials are needed to assess the efficacy of the pharmacologic and nonpharmacologic therapeutic modalities. In addition, studies of longer-term follow-up are needed to determine the prognostic benefit of these agents. Prior pharmacologic probe trials in WISE subjects have demonstrated that low-dose estrogen therapy was associated with a reduction in symptoms, but not ischemia [23]. In the WISE cohort trial women who received high dose quinapril had improved CFR after 16 weeks compared to the placebo group. In addition, the quinapril group also had improvement in angina symptoms based on the Seattle Angina Questionnaire [22], while add-on aldosterone inhibition did not result in further detectable improvements in coronary microvascular function as measured by CFR, nor did it improve endothelial dysfunction. A pilot study of ranolazine showed improved symptoms in women with angina and evidence of ischemia but no obstructive CAD, and patients with low CFR demonstrated improved CFR with treatment [24]. A similar sized study showed some improvement with symptoms but no effect on coronary microvascular function [25]. A recent large randomized trial of two-week course of ranolazine vs. placebo found no difference in symptoms or myocardial perfusion reserve [26]. However, in the prespecified subset with reduced CFR improvement in angina and myocardial perfusion was noted. None of the clinical trials to date have been of sufficient size or duration to assess clinical events. Given the fact that the presence of coronary vascular dysfunction is consistent in a similar population one decade later, with its significant relationship between measures at baseline and adverse outcomes during follow-up, a greater focus on treatment options to improve outcomes seems prudent.

A recently reported clinical trial randomized patients with symptoms of ischemia but no significant obstructive coronary artery disease to coronary reactivity study and stratified medical therapy versus standard of care alone [27]. The CorMica trial suggested that both testing for coronary vasospasm and microvascular dysfunction, which then guided medical therapy, resulted in improved angina as evidenced by an improvement in patient's Seattle Angina Questionnaire as well as their quality of life. The study also used fractional flow reserve measurements but this did not influence treatment choices.

## 5. Conclusions

Our study shows that the later WISE-CVD cohort of women with symptoms and signs of ischemia but without obstructive CAD undergoing CRT exhibits both an endothelial and a microvascular dysfunction prevalence and severity similar to that found in the earlier original WISE cohort. Thus, a decade later, in a similar population of women, even with fewer traditional risk factors, the degree of coronary vascular dysfunction remains very similar. Because these abnormalities have been linked to a risk for major adverse cardiac events, clinical trials targeting these abnormalities are indicated.

## Figures and Tables

**Table 1:** Baseline characteristics of the original WISE and subsequent WISE-CVD cohorts.

	WISE 1997-2001 n=181	WISE-CVD 2009-2011 n=235	p
Age (mean)	54 ± 10	54 ± 12	0.96
White (%)	83	74	0.040
Education (High School or more) (%)	77	97	*<0.0001*
Education (College or more) (%)	11	45	*<0.0001*
History of:		Hormone replacement therapy	
Hormone replacement therapy (%)	57	46	*0.026*
Diabetes (%)	17	12	0.11
Hyperlipidemia (%)	46	20	*<0.0001*
Hypertension (%)	52	40	*0.013*
Smoking (%)	56	46	*0.036*
Current smoker (%)	20	6	*<0.0001*
Family history of coronary heart disease (%)	62	48	*0.004*
Body mass index (mean)	31.2 ± 7.5	30.4 ± 8.5	0.30
Systolic blood pressure (mean)	134 ± 19	126 ± 18	*<0.0001*
Diastolic blood pressure (mean)	77 ± 10	69 ± 11	*<0.0001*
Arterial pressure (mean)	96 ± 11	88 ± 11	*<0.0001*
Total cholesterol (mean)	184 ± 44	182 ± 40	0.82
Low-density lipoprotein cholesterol (mean)	106 ± 38	100 ± 33	0.14
High-density lipoprotein cholesterol (mean)	52 ± 12	60 ± 17	*<0.0001*
Triglycerides (median [IQR])	103 (70, 160)	94 (70, 130)	0.17
Fasting blood sugar (mean)	103 ± 42	96 ± 28	0.07
Functional capacity (DASI) (mean)	19 ± 15	29 ± 20	*<0.0001*
Metabolic syndrome (%)	46	22	*<0.0001*
Medications last 3 months:			
Beta blockers (%)	26	25	0.74
Statins (%)	14	35	*<0.0001*
ACE inhibitors (%)	18	20	0.54
Any lipid lowering (%)	18	36	*<0.0001*
Any antihypertensive (%)	55	46	0.059
Any psychotropic (%)	31	33	0.60

IQR=interquartile range; DASI: Duke Activity Status Inventory, where higher is better functional capacity; ACE = angiotensin-converting enzyme.

**Table 2:** A comparison of coronary reactivity measures in the original and contemporary WISE cohorts.

	1997-2001	2009-2011	p*∗*
Endothelial-Independent Coronary Reactivity (CFR-Ado)	n=162	n=222	
Mean CFR-Ado	2.6 ± 0.8	2.7 ± 0.6	0.35
Median (IQR)	2.5 (2.1, 3.0)	2.6 (2.2, 2.9)	0.26
% with CFR < 2.5	48	40	0.12
% with CFR <2.32	36	31	0.27

Endothelial-Dependent Reactivity (Δ Diam to ACH)	n=107	n=162	
Mean Δ Diam	1.6 ± 12.8	0.2 ± 10.0	0.34
Median (IQR)	2.6 (-6.4, +10.0)	-0.6 (-6.4, +6.5)	0.14
% with Δ Diam ≤ 0% (abnormal)	44	54	0.10

Endothelial-Independent Reactivity (Δ Diam to NTG)	n=113	n=170	
Mean Δ Diam	9.8 ± 13.5	9.7 ± 13.0	0.94
Median (IQR)	8.3 (2.8, 17.1)	7.6 (1.3, 18.8)	0.87
% with Δ Diam ≤ 20% (abnormal)	83	78	0.28

*∗* p-values by t-test, Kruskal-Wallis test, and Chi Square, where appropriate.

CFR-Ado = coronary flow reserve in response to adenosine; IQR=inter-quartile range; ACH = acetylcholine; NTG = nitroglycerin.

## Data Availability

The fully anonymized WISE dataset is available to the public via the Biologic Specimen and Data Repository Information Coordinating Center web site at https://biolincc.nhlbi.nih.gov/studies/wise/?q=WISE.
